# Implementation of a Web-Based Application (Wellhealth) for Osteoporosis Medication Management in Older Adults: Prospective Feasibility Study

**DOI:** 10.2196/86067

**Published:** 2026-06-10

**Authors:** Chantas Mahaisavariya, Kittipoom Supamontri, Natcha Limtrakun, Apiwat Fuangfoo, Palita Sakunma

**Affiliations:** 1 Department of Orthopedic Surgery Golden Jubilee Medical Center, Faculty of Medicine Siriraj Hospital, Mahidol University Nakhon Pathom Thailand; 2 School of Business Administration Bangkok University Pathum Thani Thailand; 3 Invitrace Company Limited Bangkok Thailand

**Keywords:** feasibility study, mobile applications, osteoporosis, remote patient monitoring, telemedicine

## Abstract

**Background:**

Osteoporosis is a major global health challenge, but treatment uptake and long-term adherence remain low, raising the risk of future fractures. Barriers to effective care include low patient awareness, financial constraints, and challenges with ongoing monitoring and follow-up. Although mobile health and telemedicine tools can support chronic disease management, many osteoporosis apps lack clinical validation and structured medication management features.

**Objective:**

This study aimed to evaluate the feasibility of implementing a newly developed web-based application, Wellhealth, to support osteoporosis treatment management. A secondary objective was to explore potential associations between patient demographic characteristics and the frequency of in-app medication logging over a 1-year period.

**Methods:**

We conducted a feasibility study at the Golden Jubilee Medical Center, Mahidol University, between January 2023 and March 2025. Participants were recruited using convenience sampling during routine outpatient osteoporosis clinic visits. Eligible patients with a primary osteoporosis diagnosis used the Wellhealth web-based system, including the Assisted Liaison Service feature, over a 1-year period. Weekly in-app reports of medication use, satisfaction, and related outcomes were analyzed. Feasibility outcomes were summarized using descriptive statistics. Potential associations between participant characteristics and consistent in-app medication logging were explored using univariable and multivariable logistic regression analyses. Differences between in-app medication logging rates and medication possession ratio values were summarized using medians and IQRs.

**Results:**

We enrolled 32 participants with a mean age of 71 (range 58-91) years. The average in-app medication logging rate was 62.38% (SD 27.4%) for antiosteoporosis medications and 64.67% (SD 34.5%) for calcium supplementation. Vitamin D logging data were available for 27 participants, with an average logging rate of 68.23% (SD 31.6%). Overall satisfaction with the application was high, with 49% (15/32) of participants reporting high satisfaction, 42% (14/32) good, and 9% (3/32) average. User interaction increased markedly between the first and third quarters of 2023 before stabilizing through the second quarter of 2024. In multivariable analysis, consistent calcium logging was the only factor independently associated with higher antiosteoporosis logging rates (*P*=.02). Although younger age was associated with higher logging in univariable analysis (*P*=.01), this was no longer significant after multivariable adjustment (*P*=.12).

**Conclusions:**

Use of the Wellhealth system was feasible in this small cohort, with consistent medication logging, high user acceptability, and sustained digital engagement. Only consistent calcium logging was independently associated with higher antiosteoporosis medication tracking rates. Larger studies are needed to assess the app’s clinical effectiveness and impact on long-term outcomes.

## Introduction

Osteoporosis is a major global health concern, particularly as aging populations continue to grow, increasing both morbidity in older adults and mortality following fragility hip fractures [1-3]. Even after successful treatment of a hip fragility fracture, fewer than 50% of patients regain their prefracture level of mobility, with many requiring ongoing caregiver support, resulting in substantial health care and economic burdens [4]. Although osteoporosis treatment has been shown to improve quality of life and reduce the risk of fragility fractures in older adults [5], treatment rates remain low worldwide [6]. A recent Asia-Pacific regional audit reported an overall osteoporosis treatment rate of approximately 40.3% across the region [7]. In the United Kingdom, the treatment rate in 2021 was estimated at around 33%, leaving a treatment gap of approximately 66% [8]. Similarly, a recent retrospective study in the United States found that fewer than 20% of patients with a previous fragility fracture received osteoporosis treatment [9].

Improving osteoporosis treatment rates remains both difficult and essential for reducing the burden of fragility fractures. A previous study conducted in Thailand identified limited patient awareness as one of the main barriers to treatment uptake [10]. Long-term medication adherence is also affected by financial pressures and practical challenges related to ongoing monitoring and follow-up. Increasing awareness and maintaining close patient monitoring are therefore important components of improving treatment uptake and effectiveness [10].

With the growing use of telemedicine and mobile health technologies, several studies have explored the use of remote patient monitoring applications to improve outcomes in chronic disease management [11-14]. A recent meta-analysis suggested that some digital applications may help improve treatment management and outcomes in patients with osteoporosis [15]. However, most existing applications still lack robust evidence of clinical effectiveness and rarely include structured medication management features [15].

To help address this treatment gap, the authors developed a web-based application designed to support remote patient monitoring and clinical treatment management [16]. This study aimed to evaluate the feasibility and usability of the application in supporting the daily management of patients with osteoporosis. A secondary objective was to explore potential associations between patient demographic characteristics and the frequency of in-app medication logging. The findings from this study will be used to guide the development of a newer version of the application for evaluation in a future large-scale efficacy trial.

## Methods

### Study Design

This study was designed as a feasibility study evaluating a non–medical-device intervention conducted at the Golden Jubilee Medical Center, Mahidol University, between January 2023 and March 2025. The study population consisted of patients diagnosed with primary osteoporosis who received treatment and follow-up care through the osteoporosis clinic.

### Ethical Considerations

Ethical approval for the study was obtained from the Siriraj Institutional Review Board, Faculty of Medicine Siriraj Hospital, Mahidol University, prior to study initiation (Si-869/2022). All participants were informed about the study procedures, including the potential risks and benefits, and provided written informed consent before enrollment. Participation involved no additional costs to the participants, and no financial compensation was provided.

To protect participant privacy, all data used for research purposes were deidentified prior to analysis. Personal registration information required for the clinical operation of the application was retained within the operational system solely to maintain linkage between participants, their treating hospital, and their physician. Access to the operational dashboard and study database was restricted to authorized personnel only. All records stored on the Wellhealth server—including medication confirmation data, symptom reports, quality-of-life assessments, and communication logs—were accessible exclusively through password-protected interfaces. Data handling procedures complied with institutional research governance and confidentiality policies.

### Participants and Recruitment

The inclusion criterion for the study was a diagnosis of primary osteoporosis treated at our institution, regardless of whether the patient had a prior fragility fracture. The diagnosis of osteoporosis was based on the Thailand Osteoporosis Foundation clinical practice guideline, which defines osteoporosis as any of the following: a fragility fracture of the hip or spine regardless of bone mineral density (BMD); a BMD T-score of –2.5 or lower at any hip or spine site in patients without prior fracture; or a fragility fracture at a site other than the hip or spine in patients with a BMD T-score between –1.0 and –2.5. In accordance with hospital protocol, BMD was assessed using standard dual-energy x-ray absorptiometry. Both treatment-naive patients and those already receiving osteoporosis treatment were eligible for inclusion. Patients were excluded if they had chronic kidney disease with an estimated glomerular filtration rate of ≤25 mL/min/1.73 m^2^, an allergy to antiosteoporosis medications, an inability to read or write, an inability of either the participant or caregiver to use or access a smartphone or the application, or cognitive impairment that prevented completion of the study questionnaire. Older adults who were unfamiliar with smartphone use could still participate if their primary caregiver agreed to use the application on their behalf. In these cases, only the patient with osteoporosis was considered a study participant, while the caregiver was not formally enrolled in the study. To meet eligibility requirements, the accompanying caregiver had to be a relative and serve as the participant’s full-time caregiver. This approach was considered practical within the Thai context, where caregivers are commonly adult children or relatives living in the same household.

A convenience sampling strategy was used for participant recruitment. Participants were recruited through the osteoporosis clinic at the study site, where secondary causes of osteoporosis were excluded through clinical assessment and investigation. Eligible patients were identified during routine outpatient appointments. The treating orthopedic surgeon (CM) introduced the study during the clinical consultation, explained the study objectives, and assessed each patient’s initial interest in participation. To minimize the risk of coercion bias and reduce any perceived obligation to participate when approached by the treating physician, patients who expressed interest were subsequently referred to a clinic nurse who was not directly involved in their clinical care. The nurse then conducted the formal informed consent process, including a detailed explanation of the study procedures, potential risks and benefits, and the voluntary nature of participation. Only patients who provided written informed consent to the nurse were enrolled in the study.

### Wellhealth Digital Platform

After enrollment, participants were instructed to use the Wellhealth system, including the Assisted Liaison Service feature, for ongoing osteoporosis monitoring. Patients and caregivers registered their personal information within the application, with all data stored anonymously in the database. Patients or their caregivers entered details of osteoporosis medications and supplements, including dosage, frequency, and administration time. The application then generated reminders for scheduled medication doses. Following each dose, patients or caregivers confirmed medication intake through an in-app confirmation button, which automatically recorded and transmitted the data to the database.

### Wellhealth System Architecture

**Figure 1:**
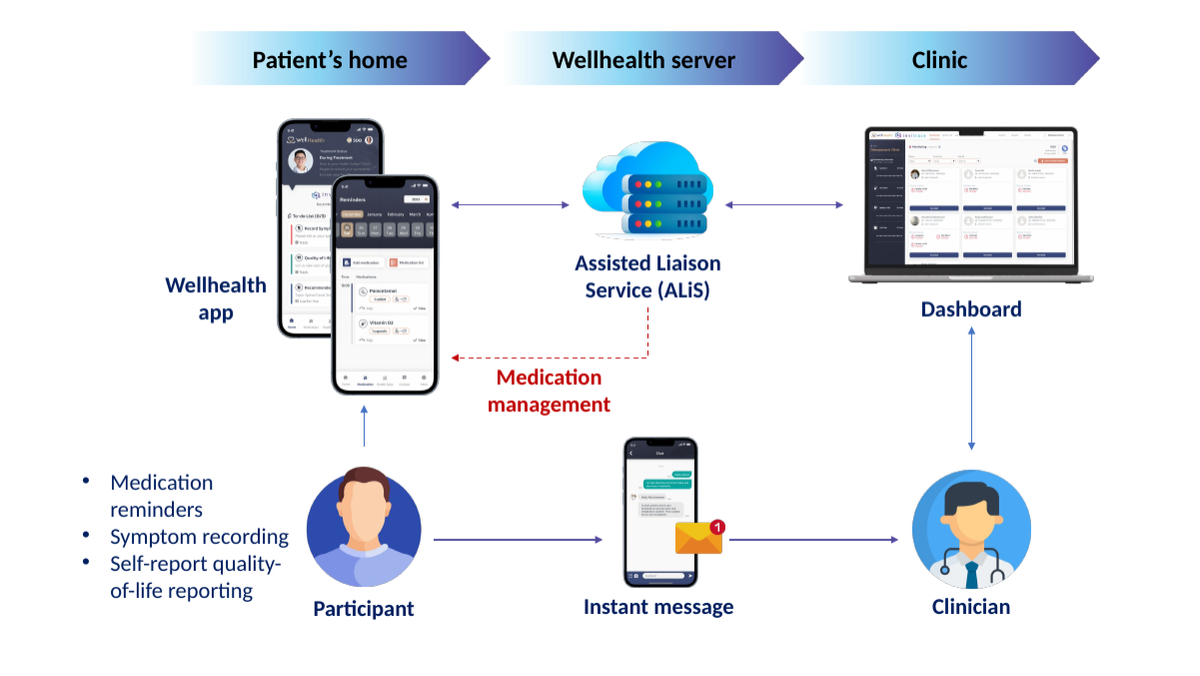
Architecture of the Wellhealth system. The Wellhealth system consists of 3 interconnected components: a patient-facing mobile app used for medication reminders and self-reporting, a central server incorporating the Assisted Liaison Service to enable bidirectional data transfer, and a clinician-facing dashboard. The dashboard allows the care team to monitor longitudinal patient data, medication records, and flagged health-related events.

As illustrated in Figure 1, the Wellhealth system consisted of 3 integrated components: a patient-facing mobile app used in the home setting, a central server supporting the Assisted Liaison Service feature, and a clinician-facing dashboard used by the care team. The mobile app was installed on the smartphone of either the participant or caregiver and served as the primary interface for medication reminders, symptom recording, self-assessment of quality of life (described in the Data Collection and Follow-Up section), and communication with the care team. The central server received and stored records generated by the application and enabled bidirectional data transfer between the patient application and the clinician dashboard. The clinician dashboard allowed authorized health care personnel to review longitudinal patient-reported information, medication confirmation records, and flagged health-related events.

Following enrollment, each participant was registered within the Wellhealth system and linked to their treating hospital, clinic, and physician. The application supported medication scheduling by allowing participants, caregivers, or study staff to enter details including medication name, dosage, frequency, route of administration, administration instructions, and reminder times. Once configured, the application generated scheduled medication reminders automatically. The application used a single-action design for daily medication confirmation, in which participants were instructed to press a designated button only after taking their medication. No options were provided to record a “skipped” or “snoozed” status. Medication confirmation records were transmitted to the server and displayed on the clinician dashboard as part of the medication monitoring system.

In addition to medication management, the application supported routine health monitoring. The home screen displayed pending tasks and upcoming scheduled activities. Participants were prompted to record symptom-related information at predefined intervals and complete monthly self-reported quality-of-life assessments. The application also included a health results section summarizing symptom severity, potential medication-related adverse effects, quality-of-life trends, and other health-related data.

The communication feature enabled direct contact between participants and the care team. Through the in-app messaging system, participants could send text messages to their physician or other designated health care personnel. However, participants were instructed to seek standard emergency medical care for urgent situations rather than relying solely on the application.

For the purposes of this study, the Wellhealth system functioned as a monitoring and communication platform rather than an automated treatment decision tool. All clinical management remained under physician supervision, and treatment decisions continued to follow routine clinical care practices.

### Data Collection and Follow-Up

The application generated weekly health surveillance prompts and collected information related to adverse events and quality-of-life self-assessments. Because completing lengthy standardized questionnaires on a mobile device was considered potentially burdensome for participants, the development team created a brief self-assessment instrument specifically for this feasibility study. The instrument evaluated 5 domains: physical health, work capacity, financial status, mental health, and social health and relationships. Each domain consisted of 3 short subquestions. Responses were scored using a 5-point scale, with higher scores indicating better perceived quality of life. The questionnaire was originally developed in Thai, while the English version is provided in Multimedia Appendix 1. The application’s instant messaging feature also allowed participants to contact the care team regarding urgent issues, such as suspected medication allergies or falls.

Patient satisfaction with the application was assessed weekly using a 5-point rating scale, where 5 represented the highest level of satisfaction and 1 the lowest. Overall satisfaction ratings were aggregated and reported as monthly average scores. Satisfaction data were transmitted to the clinician dashboard for monitoring and analysis.

Participants attended routine osteoporosis clinic visits with their physician at baseline and again at 6 and 12 months. Additional visits could be arranged either by appointment or on a walk-in basis if participants experienced changes in symptoms or other health-related concerns. Laboratory investigations and physical examinations were conducted as part of standard clinical care, and study staff regularly reminded participants to continue using the application.

### Outcomes

The primary outcome of this study was the feasibility of implementing the Wellhealth application, assessed through patient usage, acceptability, and retention over a 1-year period. Use of the application’s core function was evaluated using in-app medication logging rates across 3 categories: calcium, vitamin D, and antiosteoporosis medications. Consistency of application use was also assessed using clinical thresholds commonly applied in medication persistence research. Sustained users were defined as participants who continued interacting with daily prompts while allowing gaps of up to 30 days, in line with previously established medication persistence standards [17]. App engagement was categorized as excellent (≥80%), good (60%-79%), acceptable (50%-59%), or poor (<50%). Optimal logging behavior was defined as responding to at least 80% of prompts, based on engagement thresholds adapted from prior medication persistence studies [17-19].

Application acceptability was evaluated using patient satisfaction scores collected monthly and cumulatively at the end of the study period. Retention and overall digital engagement were assessed using the frequency of participant interactions recorded within the application database, along with the number of online consultations conducted per quarter throughout the 1-year follow-up period, excluding medication logging prompts. In addition to the primary feasibility outcomes, self-reported quality of life was analyzed as a preliminary descriptive outcome and categorized according to the percentage of the maximum possible score: 80%-100% as excellent, 60%-79% as good, 40%-59% as moderate, 20%-39% as fair, and below 20% as poor.

A secondary objective of the study was to explore potential associations between patient characteristics and consistent in-app medication logging behavior. Because the application was specifically developed to support osteoporosis treatment management, logging of antiosteoporosis medications was considered the most clinically important behavioral outcome. The analysis, therefore, focused on identifying demographic characteristics and broader patterns of application engagement associated with higher logging rates for antiosteoporosis medications.

We also evaluated discordance between the medication possession ratio (MPR) derived from hospital medical records and in-app medication logging rates measured over the same time period. Within the Thai prescription system, physicians prescribe medications during hospital visits, and patients collect the medications immediately afterward from the hospital pharmacy. Although this process confirms medication collection, it does not verify actual medication administration. In-app medication logging instead captures patient- or caregiver-reported daily intake recorded within the application. The 2 measures, therefore, reflect different aspects of medication-use behavior, and each is subject to its own sources of error. Discordance between these measures was assessed across 3 medication categories: calcium, vitamin D, and antiosteoporosis medications.

Side effects and concerning symptoms reported by participants were recorded in real time through the application, with automated alert notifications transmitted to the clinician dashboard. Participants were permitted to continue using the application beyond 1 year; however, only data collected during the first year were included in the study analysis.

### Statistical Analysis

Statistical analyses were performed using SPSS Statistics (IBM Corp). Demographic variables were summarized using counts and proportions, while age was reported as mean and SD. Categorical variables, including patient expectations, treatment affordability, medical reimbursement status, and financial status, were expressed as percentages.

The in-app medication logging rate was calculated by dividing the number of logged dose confirmations by the total number of medication reminders sent through the application. This calculation was performed separately for all prescribed medications, including calcium, vitamin D, and antiosteoporosis agents.

Patient satisfaction with application use was assessed weekly through an in-app rating prompt and aggregated into monthly average scores. Each participant, therefore, contributed 13 monthly assessment points beginning from the initiation of application use. Because participants were enrolled at different time points, assessment schedules varied between individuals. However, each participant was evaluated monthly relative to their own enrollment date, resulting in 13 assessments per participant. Satisfaction scores were then aligned chronologically according to assessment month (months 1-13) across all participants, and the average satisfaction score for each time point was reported as a percentage according to the predefined satisfaction categories. Overall satisfaction was calculated by aggregating scores across all assessment periods.

Patient-application interaction was quantified by counting the number of recorded interactions, excluding medication logging prompts. Interaction frequency was analyzed across predefined quarterly intervals over the 1-year follow-up period.

Potential factors associated with higher antiosteoporosis medication logging rates were explored using logistic regression analysis. Univariable analyses were first performed to identify factors individually associated with the outcome, followed by multivariable logistic regression to determine independent associations after adjustment for potential confounding variables. Statistical significance was defined as *P*<.05. Side effects were reported descriptively as the number of observed cases.

Discordance between the MPR and the in-app medication logging rate was calculated using the following formula: {Discordance} = {In-app logging rate} – {MPR}

The frequency distribution of discordance values within each range was presented using histograms, and discordance across all 3 medication categories was summarized using medians and IQRs.

As this study was designed as a feasibility study, a minimum sample size of 30 participants was considered sufficient to identify preliminary trends related to the feasibility of implementing the intervention.

## Results

### Participant Flow and Baseline Characteristics

Participant recruitment began following institutional review board approval in January 2023. During the first quarter of 2023, a total of 12 patients were enrolled, followed by an additional 6 participants in the second quarter. One participant died from a chronic underlying medical condition during the fifth month after enrollment, as reported by the caregiver through the in-app messaging system. Two participants were enrolled during the third quarter of 2023, followed by 6 more in the fourth quarter. The final 6 participants were enrolled during the first quarter of 2024.

**Figure 2:**
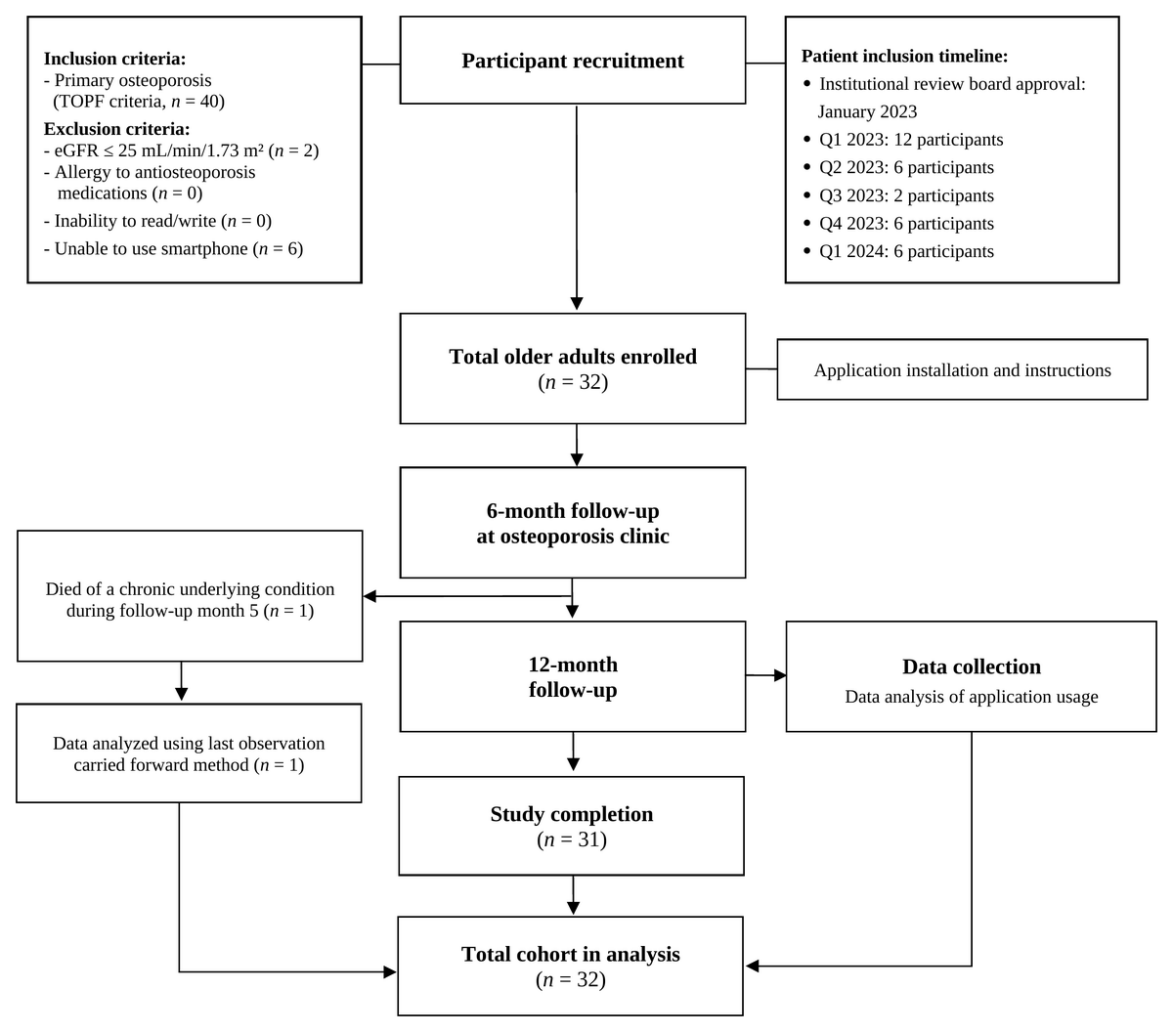
Flow diagram of the study process in the pilot prospective cohort study evaluating the Wellhealth web-based application among patients with osteoporosis at the Golden Jubilee Medical Center, Thailand, conducted between January 2023 and March 2025. Patients meeting the inclusion criteria of the Thailand Osteoporosis Foundation were enrolled and provided with the Wellhealth application, including installation and usage instructions. Exclusion criteria are listed on the left side of the figure. Of the 32 enrolled older adults, 1 participant died during follow-up because of an unrelated chronic medical condition. Data from this participant were analyzed using the last-observation-carried-forward method. eGFR: estimated glomerular filtration rate; TOPF: Thailand Osteoporosis Foundation.

In total, 32 older adults were enrolled in the study. Data from the participant who died during follow-up were analyzed using the last observation carried forward method. The remaining 31 participants completed the full 1-year follow-up period. All analyses were performed using the complete cohort of 32 participants (Figure 2).

The mean participant age was 71 (range 58-91) years. Additional demographic characteristics are presented in Table 1.

**Table 1:** Baseline characteristics of participants (n=32).

Characteristic		Study sample (n=32)
**Age (years)**		
	Minimum-maximum	58-91
	Mean (SD)	71 (8.5)
**Patient expectation, n (%)**		
	Cure	28 (87.5)
	Control	2 (6.3)
	Comfort	1 (3.1)
	N/A^a^	1 (3.1)
**Patient affordability, n (%)**		
	Best standard care	9 (28.1)
	Extra cost with a limited budget	22 (68.8)
	Extra cost with unlimited budget	1 (3.1)
**Payment readiness^b^** **, n (%)**		
	Not at all	2 (6.3)
	Slightly	1 (3.1)
	Sometimes	17 (53.1)
	Mostly	11 (34.4)
	Extremely	1 (3.1)
**Payment options^b^** **, n (%)**		
	Not at all	1 (3.1)
	Slightly	1 (3.1)
	Sometimes	19 (59.4)
	Mostly	9 (28.1)
	Extremely	2 (6.3)
**Patient coverage scheme, n (%)**		
	Direct disbursement	21 (65.6)
	Social Security	1 (3.1)
	Cash	10 (31.3)

^a^N/A: not available.

^b^Payment readiness and preferred payment options were assessed using a 5-point ordinal scale.

### In-App Medication Logging Rate of Prescriptions

The overall in-app medication logging rate was 62.38% (SD 27.4%) for antiosteoporosis medications and 64.67% (SD 34.5%) for calcium supplementation. Vitamin D logging data were available for 27 participants, as supplementation had been temporarily discontinued in patients with elevated serum vitamin D levels. Among these participants, the in-app medication logging rate for vitamin D supplementation was 68.23% (SD 31.6%; Table 2).

Using an in-app medication logging rate of ≥80% as the threshold for optimal consistency, the proportion of participants meeting this criterion was 34.4% (11/32) for antiosteoporosis medications, 43.8% (14/32) for calcium supplementation, and 48.2% (13/27) for vitamin D supplementation. These frequencies were compared descriptively with baseline medication possession patterns obtained from hospital prescription records to provide additional context regarding patients’ digital medication logging behavior relative to clinical dispensing records (Table 3).

**Table 2:** In-app medication logging rate at 1 year.

Prescription	Participants, n	In-app medication logging (%)^a^, mean (SD)	Consistent^b^, n (%)	Nonconsistent^c^, n (%)
Antiosteoporosis	32	62.38 (27.4)	11 (34.4)	21 (65.6)
Vitamin D^d^	27	68.23 (31.6)	13 (48.2)	14 (51.8)
Calcium	32	64.67 (34.5)	14 (43.8)	18 (56.2)

^a^In-app medication logging was calculated from self-recorded in-app confirmations across 12 months.

^b^Consistent was defined as in-app medication logging ≥80%.

^c^Nonconsistent was defined as in-app medication logging <80%.

^d^Vitamin D data were available for n=27 because supplementation was paused for elevated serum vitamin D.

**Table 3:** Distribution of in-app medication logging by category at 1 year.

Medication^a^		Value, n (%)
**Antiosteoporosis (n=32)**		
	Excellent (≥80%)	11 (34.4)
	Good (60%-79%)	2 (6.2)
	Acceptable (50%-59%)	14 (43.8)
	Poor (<50%)	5 (15.6)
**Vitamin D^b^** **(n=27)**		
	Excellent (≥80%)	13 (48.2)
	Good (60%-79%)	2 (7.4)
	Acceptable (50%-59%)	5 (18.5)
	Poor (<50%)	7 (25.9)
**Calcium (n=32)**		
	Excellent (≥80%)	14 (43.8)
	Good (60%-79%)	1 (3.1)
	Acceptable (50%-59%)	8 (25)
	Poor (<50%)	9 (28.1)

^a^Category thresholds for in-app medication logging were defined as follows: excellent, ≥80%; good, 60%–79%; acceptable, 50%–59%; and poor, <50%.

^b^Vitamin D data were available for n=27 because supplementation was paused for elevated serum vitamin D.

### App Engagement, Retention, and Acceptability

**Figure 3:**
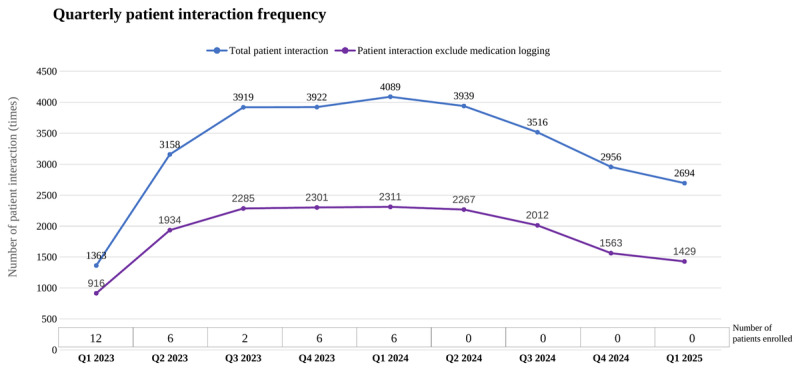
Quarterly counts of patient-application interactions recorded by the Wellhealth system during the study period. The blue line represents the total number of patient interactions recorded per quarter, including both in-app medication logging confirmations and all other application activity. The purple line represents nonlogging engagement, calculated after excluding medication logging confirmations. The number of participants newly enrolled during each quarter is displayed beneath the x-axis. Overall activity increased markedly from Q1 to Q3 2023, remained relatively stable from Q3 2023 through Q2 2024, and gradually declined toward the end of the study period.

Figure 3 illustrates trends in patient-application interaction over the study period. Total interactions increased substantially during the early phase of the study, rising from 1363 interactions in Q1 2023 (January-March 2023) to 3919 interactions in Q3 2023 (July-September 2023). Interaction frequency then stabilized, reaching a peak of 4089 interactions in Q1 2024 before gradually declining toward the end of the observation period. Across the full study duration, participants generated a total of 29,556 interactions.

To better evaluate broader application engagement, medication logging interactions were excluded from this analysis. When considering only nonlogging interactions, total engagement was 17,018 interactions, with a peak of 2311 interactions observed in Q1 2024, as illustrated by the lower trendline in Figure 3. Nonlogging interactions included survey responses, in-app messaging, and routine use of the application, although these interaction types were not analyzed separately in this feasibility study. Quarterly nonlogging engagement increased from 916 interactions in Q1 2023, when 12 participants had been enrolled, to a peak of 2311 interactions in Q1 2024, when the full cohort of 32 participants had been enrolled. Engagement then remained relatively stable in Q2 2024 (2267 interactions) before gradually declining to 2012 interactions in Q3 2024, 1563 in Q4 2024, and 1429 in Q1 2025 (Figure 3).

**Figure 4:**
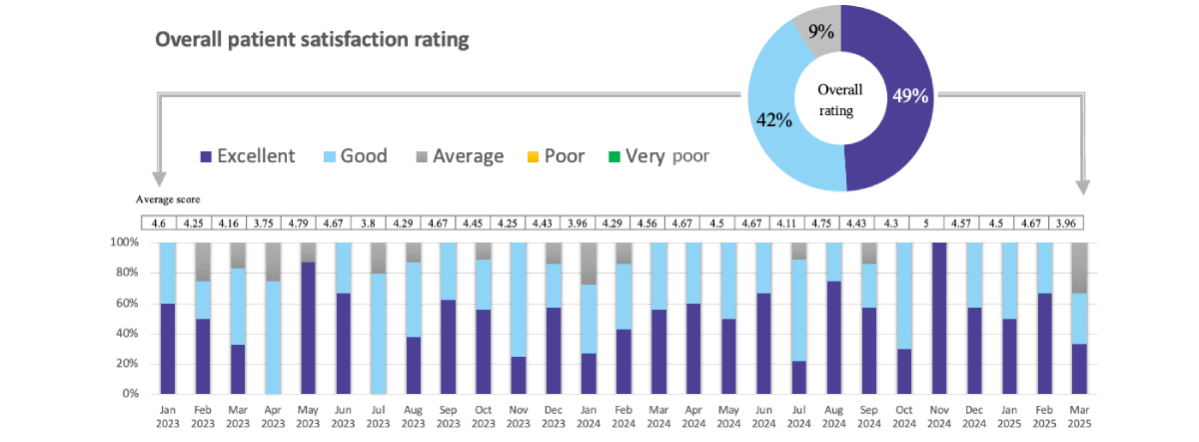
Distribution of patient satisfaction ratings over 12 months. Ratings were collected weekly through the application during follow-up and are presented as category frequencies across the study period (overall distribution shown in the pie chart and monthly distributions shown in the stacked bar chart). Most ratings were classified as excellent (15/32, 49%) or good (14/32, 42%), while 9% (3/32) were rated as average. No ratings were classified as poor or very poor.

Patient satisfaction was summarized using monthly average scores, with the overall satisfaction rate calculated as the mean across the entire study period. Most satisfaction ratings were classified as excellent (15/32, 49%), followed by good (14/32, 42%) and average (3/32, 9%). Monthly satisfaction trends are presented in Figure 4.

**Figure 5:**
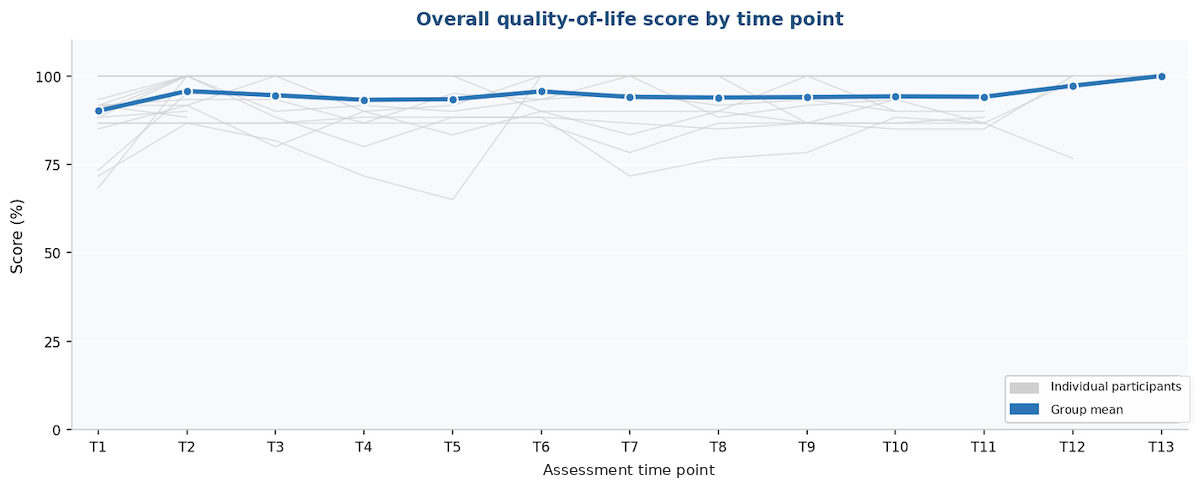
Overall quality-of-life assessment throughout the study period. Self-reported quality-of-life scores across the 13 monthly assessment time points (T1-T13). Thin grey lines represent individual participant trajectories, while the bold blue line represents the cohort mean. Scores were calculated as a percentage of the maximum possible total across the 5 assessed domains: physical health, work and productivity, financial well-being, mental health, and social health. T1=baseline; T2-T13=follow-up months 1-12.

As a preliminary descriptive outcome, self-reported quality of life was monitored longitudinally across the 5 domains described in the Methods section. Overall quality-of-life scores consistently exceeded 80% throughout follow-up. Physical health was the lowest-scoring domain, with scores ranging from 73.3% to 100% and a mean score of 80.87 (SD 7.5; Table 4 and Figure 5).

Across the cohort, 200 of 416 expected monthly quality-of-life self-assessments were completed (32 participants × 13 monthly assessment points), corresponding to an overall response rate of 48.1%. Six (18.8%) participants did not submit any quality-of-life assessments during the study period, while the remaining 26 (81.3%) participants completed at least 1 assessment. Among these active responders, completion rates declined progressively over time: 26/26 (100%) at baseline (T1), 21/26 (80.8%) at T2, 16/26 (61.5%) at T6, 15/26 (57.7%) at T9, 13/26 (50%) at T11, 9/26 (34.6%) at T12, and 2/26 (7.7%) at T13.

A response rate for the weekly satisfaction prompt could not be calculated because the application database retained only aggregated monthly satisfaction scores rather than individual prompt completion records.

**Table 4:** Quality-of-life domain scores by monthly assessment time point (T1-T13).

Time point	Responders, n (%)^a^	Physical health (%)^b^, mean (SD)	Work and productivity (%)^b^, mean (SD)	Financial well-being (%)^b^, mean (SD)	Mental health (%)^b^, mean (SD)	Social health (%)^b^, mean (SD)	Overall (%)^b^, mean (SD)
T1 (baseline)	26 (81.3)	74.36 (26.45)	96.47 (12.51)	89.74 (23.25)	90.06 (19.86)	100.00 (0.00)	90.13 (10.16)
T2	21 (65.6)	83.73 (25.20)	100.00 (0.00)	96.83 (14.55)	100.00 (0.00)	98.02 (9.09)	95.71 (5.44)
T3	18 (56.3)	81.94 (25.76)	100.00 (0.00)	99.07 (3.93)	97.22 (8.57)	94.44 (16.42)	94.54 (7.11)
T4	17 (53.1)	77.94 (27.94)	96.57 (14.15)	95.10 (20.21)	100.00 (0.00)	96.57 (14.15)	93.24 (8.61)
T5	17 (53.1)	78.43 (28.12)	100.00 (0.00)	97.06 (12.13)	97.55 (10.11)	94.12 (16.86)	93.43 (9.40)
T6	16 (50.0)	80.21 (25.80)	100.00 (0.00)	100.00 (0.00)	100.00 (0.00)	97.92 (8.33)	95.62 (5.37)
T7	16 (50.0)	80.73 (27.84)	97.40 (10.42)	100.00 (0.00)	94.27 (13.17)	97.92 (8.33)	94.06 (9.27)
T8	15 (46.9)	75.56 (29.79)	96.11 (15.06)	100.00 (0.00)	97.78 (8.61)	100.00 (0.00)	93.89 (7.52)
T9	15 (46.9)	77.78 (29.49)	96.67 (12.91)	100.00 (0.00)	97.22 (10.76)	98.33 (6.45)	94.00 (7.34)
T10	15 (46.9)	73.33 (30.89)	100.00 (0.00)	100.00 (0.00)	97.78 (8.61)	100.00 (0.00)	94.22 (6.00)
T11	13 (40.6)	75.64 (32.54)	100.00 (0.00)	100.00 (0.00)	97.44 (9.24)	97.44 (9.24)	94.10 (6.72)
T12	9 (28.1)	91.67 (22.05)	100.00 (0.00)	100.00 (0.00)	94.44 (16.67)	100.00 (0.00)	97.22 (7.73)
T13	2 (6.3)	100.00 (0.00)	100.00 (0.00)	100.00 (0.00)	100.00 (0.00)	100.00 (0.00)	100.00 (0.00)
Mean	N/A^c^	80.87 (7.48)	98.71 (1.72)	98.29 (3.04)	97.21 (2.88)	98.06 (2.04)	94.63 (2.29)

^a^The “Responders, n (%)” column shows the number of participants (out of 32 enrolled) who submitted a quality-of-life self-assessment at each time point; means at later time points are based on a small number of responders and should be interpreted with caution.

^b^Values are the mean percentage of the maximum possible domain score (0%-100%) at each monthly assessment (T1=baseline; T2-T13=follow-up months 1-12). Each domain score combines 3 short subquestions rated 1-5; subquestion ratings were summed and rescaled to a 0%-100% percentage. The Overall column is the mean across the 5 domains.

^c^N/A: not applicable.

### Factors Associated With Higher Antiosteoporosis Medication Logging

The analysis explored factors potentially associated with consistent antiosteoporosis medication logging, as antiosteoporosis agents represented the primary disease-modifying treatment that the application was designed to support. Demographic variables evaluated included age, treatment expectations, financial affordability, payment options, payment readiness, and health care payment coverage scheme. Additional variables derived from application data included calcium logging consistency, vitamin D logging consistency, and self-reported quality of life.

In univariable analysis, younger age and consistent calcium logging were both positively associated with higher antiosteoporosis medication logging rates. However, in the multivariable logistic regression model, only consistent calcium logging remained independently associated with higher logging consistency (*P*=.02), while the association with younger age was no longer statistically significant (*P*=.12). Relevant effect estimates are presented in Table 5.

**Table 5:** Factors associated with higher antiosteoporosis medication logging^a^.

Variables		Univariable			
		Consistent^b^, n=11	Nonconsistent, n=21	*P* value	
Age (years), mean (SD)		66.4 (6)	73.5 (8.7)	.01	
**Vitamin D, n (%)**					.19
	Consistent	7 (63.6)	6 (28.6)		
	Nonconsistent	3 (27.3)	11 (52.4)		
	N/A^c^	1 (9.1)	4 (19)		
**Calcium, n (%)**					.03
	Consistent	8 (72.7)	6 (28.6)		
	Nonconsistent	3 (27.3)	15 (71.4)		
	N/A	0 (0)	0 (0)		
**Patient expectation, n (%)**					.69
	Cure	11 (100)	17 (81)		
	Control	0 (0)	2 (9.5)		
	Comfort	0 (0)	1 (4.8)		
	N/A	0 (0)	1 (4.8)		
**Patient affordability, n (%)**					.79
	Best standard care	4 (36.4)	5 (23.8)		
	Extra cost, limited budget	7 (63.6)	15 (71.4)		
	Extra cost, unlimited budget	0 (0)	1 (4.8)		
**Payment readiness, n (%)**					.94
	Not at all	1 (9.1)	1 (4.8)		
	Slightly	0 (0)	1 (4.8)		
	Sometimes	7 (63.6)	10 (47.6)		
	Mostly	3 (27.3)	8 (38.1)		
	Extremely	0 (0)	1 (4.8)		
**Payment options, n (%)**					.61
	Not at all	0 (0)	1 (4.8)		
	Slightly	1 (9.1)	0 (0)		
	Sometimes	7 (63.6)	12 (57.1)		
	Mostly	2 (18.2)	7 (33.3)		
	Extremely	1 (9.1)	1 (4.8)		
**Patient coverage scheme, n (%)**					.41
	Direct disbursement	9 (81.8)	12 (57.1)		
	Social Security	0 (0)	1 (4.8)		
	Cash	2 (18.2)	8 (38.1)		

^a^In multivariable analysis, age was not significantly associated with consistency (odds ratio [OR], 0.895, 95% CI 0.78-1.03; *P*=.12), whereas calcium remained significantly associated with consistency (OR 0.928, 95% CI 0.87-0.99; *P*=.02).

^b^Consistent was defined as a consistent in-app antiosteoporosis medication logging of ≥80%.

^c^N/A: not available (missing response).

### Discordance Between In-App Medication Logging and MPR

**Figure 6:**
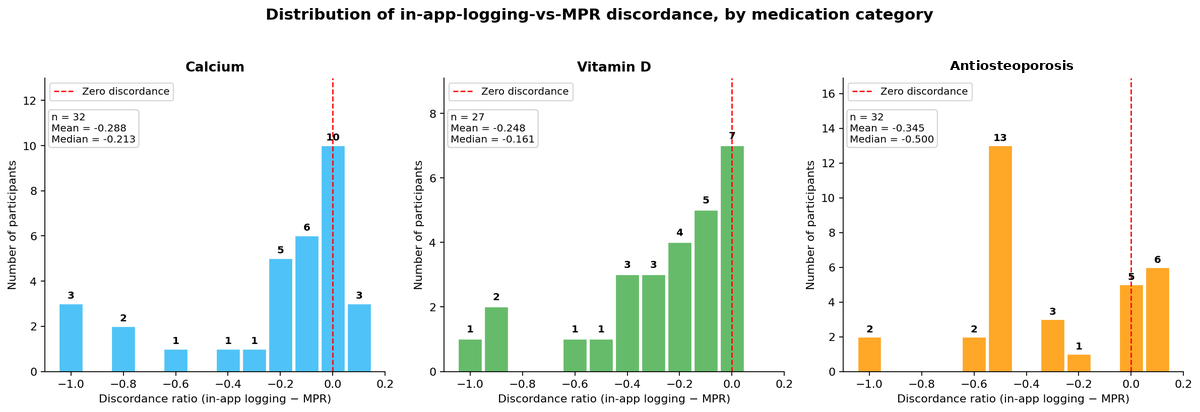
Distribution of discordance between in-app medication logging rates and medication possession ratio (MPR) values across the different medication categories. Histograms illustrating the discordance ratio between in-app medication logging rates and MPR values derived from hospital prescription records across the 3 medication categories: calcium, vitamin D, and antiosteoporosis medications. A discordance value of 0 indicates perfect agreement between the 2 measures, whereas negative values indicate that the in-app medication logging rate was lower than the MPR-derived estimate. The dashed vertical line represents the zero-discordance reference point.

Discordance between in-app medication logging rates and the MPR was evaluated across the 3 medication categories (Figure 6). Because the data were not normally distributed, results were summarized using medians and IQRs. For calcium supplementation, the median discordance was –21.3% (IQR –40.8% to –4.0%). For vitamin D supplementation, the median discordance was –16.1% (IQR –41.5% to –0.9%). Antiosteoporosis medications demonstrated the greatest discrepancy, with a median discordance of –50.0% (IQR –50.0% to –9.1%). Overall, in-app medication logging rates were consistently lower than the clinical MPR values obtained from hospital prescription records across all 3 medication categories.

### Side-Effect and Abnormal-Symptom Report

Few side effects or abnormal symptoms were reported during the study period. A total of 6 side effect events were recorded, primarily involving constipation and gastrointestinal discomfort. Four symptom alerts were generated during follow-up, including reports of dizziness, numbness in the hands or feet, gait instability, and leg cramping.

**Figure 7:**
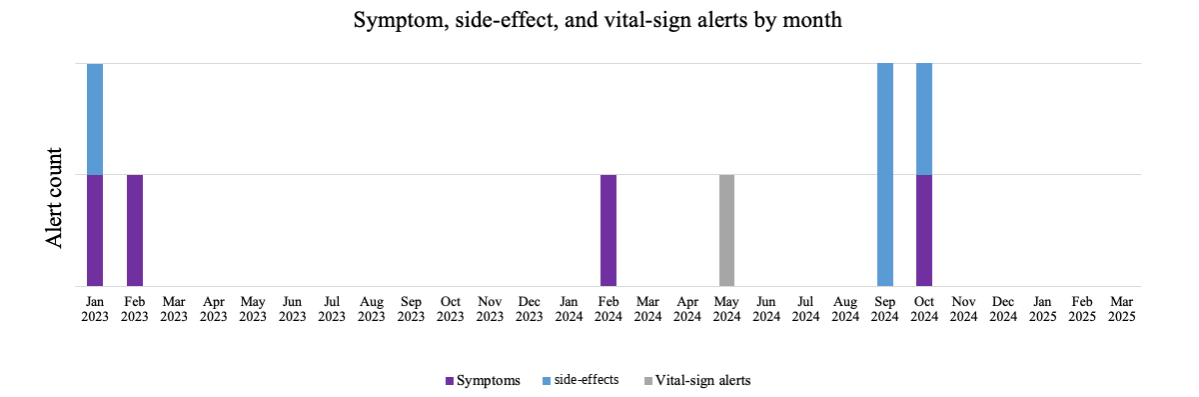
Monthly counts of symptom alerts, side-effect reports, and vital-sign alerts recorded during the study period. Bar chart showing the monthly frequency of patient-reported symptoms, side effects, and vital-sign alerts recorded by the application between January 2023 and March 2025. A total of 6 side-effect events were reported, consisting primarily of constipation and gastrointestinal discomfort. Four symptom alerts were recorded, including dizziness, numbness of the hands or feet, gait instability, and leg cramping. One vital-sign alert related to elevated blood pressure was also documented. No falls, fractures, or serious adverse events occurred during the study period.

No falls or new fractures were reported during the study period. One vital sign alert was triggered because of elevated blood pressure; however, no serious adverse events were observed throughout the study (Figure 7).

## Discussion

### Feasibility of Implementation

This study demonstrates the feasibility of implementing a remote monitoring application to support older adults receiving osteoporosis treatment. The overall mean in-app medication logging rate was approximately 60%-65% across the 3 medication categories, while 34%-48% of participants achieved the predefined consistency threshold of ≥80%. In contrast, the proportion of participants with poor logging consistency (<50%) ranged from 15% to 28% across calcium, vitamin D, and antiosteoporosis medications.

These findings suggest that most older adult participants were able to maintain ongoing digital engagement following implementation of the application, although a substantial minority experienced difficulty sustaining consistent use. Application acceptability was high, with most participants reporting good-to-excellent satisfaction levels. Retention also appeared acceptable, as reflected by overall nonlogging engagement trends, which increased markedly through the third quarter of 2023, remained relatively stable through the second quarter of 2024, and then gradually declined toward the end of the study period.

### Factors Associated With Consistent In-App Antiosteoporosis Medication Logging

Our multivariable analysis showed that consistent calcium logging was the only factor independently associated with higher antiosteoporosis medication tracking. Because calcium supplementation is a routine and foundational component of osteoporosis management, participants who regularly responded to daily calcium reminders may have developed stronger overall engagement with the application, which could in turn reinforce broader digital tracking behaviors [20].

Although younger participants initially appeared to demonstrate better logging consistency, possibly because of greater familiarity with technology or fewer cognitive barriers, this association was no longer significant after adjustment for other variables. This finding suggests that establishing a consistent daily tracking routine, such as calcium logging, may help reduce some age-related barriers to digital engagement.

Interestingly, neither medical reimbursement status nor perceived medication costs were associated with logging consistency, which differs from commonly cited assumptions regarding financial barriers to osteoporosis treatment adherence [10,21]. Further studies with larger sample sizes and dedicated clinical trials are needed to determine whether application-driven habit formation and increased disease awareness can help reduce the long-term impact of financial and logistical barriers on treatment-related behaviors.

### Discordance Between MPR and In-App Medication Logging

In-app medication logging rates were consistently lower than MPR values across all 3 medication categories. The median discordance was smaller for calcium supplementation (–21.3%, IQR –40.8% to –4.0%) and vitamin D supplementation (–16.1%, IQR –41.5% to –0.9%) than for antiosteoporosis medications (–50.0%, IQR –50.0% to –9.1%). Participants with high in-app logging consistency tended to cluster near zero discordance, whereas those with discordance values greater than 50% generally demonstrated poor logging consistency. This pattern can be summarized as follows: when participants engaged consistently with the application, their in-app logs converged with hospital-based MPR values, whereas larger discrepancies were observed predominantly in participants whose overall logging consistency was poor. These findings are descriptive and based solely on this cohort. As no internal validation, such as pill counts, was conducted, the precise reason for the discordance remains uncertain. It is unclear whether these differences reflect underlogging in the application, overestimation by MPR, or both. Additional studies are needed to clarify the source and direction of this discordance.

### Comparison With Prior Studies

Most applications identified in a recent meta-analysis primarily focused on osteoporosis education and informational support [15]. Only 3 applications—Calcium Pro, Vitamin-D Pro, and My Osteoporosis Manager—included medication tracking features [15]. Compared with these applications, the Wellhealth system integrates tracking for both prescribed osteoporosis medications and daily supplements, allowing the collection of detailed data related to logging consistency and user tracking behavior.

However, the current version of the application had several limitations. First, it did not include integrated disease education or lifestyle modification guidance. Second, although the system was designed to alert clinicians to concerning patient-reported symptoms, the response pathway relied primarily on online consultation, which may have introduced delays depending on clinician availability. Finally, because this pilot version prioritized usability for older adults, lengthy standardized quality-of-life assessment instruments were intentionally not implemented, resulting in less precise measurement of that outcome domain.

### Author Perspective: Disease Burden and Treatment Gaps

Osteoporosis often progresses without obvious symptoms, which can reduce awareness among both patients and health care providers and contribute to low treatment initiation rates [22,23]. As a result, undertreatment remains common and may lead to serious complications, including vertebral and hip fractures, highlighting the importance of public health promotion and screening programs to support earlier diagnosis and intervention [24]. Maintaining long-term treatment adherence is also challenging because osteoporosis therapy often requires prolonged treatment over many years [21]. In Thailand, Mahaisavariya et al [10] reported that antiosteoporosis treatment was frequently discontinued because of logistical barriers and patient uncertainty regarding treatment effectiveness and necessity. Improving osteoporosis management, therefore, requires greater public awareness, more efficient health care logistics, and stronger patient education regarding the disease and its treatment options [25,26].

This feasibility study suggests that a relatively simple digital intervention, consisting of mobile reminders and 1-tap in-app logging, can be implemented successfully among older adults undergoing osteoporosis treatment over a 1-year period. However, several limitations identified in this initial version of the application should be addressed before wider implementation. A notable minority of participants did not achieve the predefined logging consistency threshold, response rates for in-app surveys declined progressively during follow-up, and the current system records logging events without directly verifying actual medication administration. Addressing these limitations in a refined future version of the application, followed by larger and longer-term implementation studies, will be important for determining its true clinical impact within the broader context of remote patient monitoring research [27-29].

### Limitations

This study and the remote patient monitoring application have several important limitations. First, the sample size was small, and recruitment was constrained by limited smartphone access, poor internet connectivity, and caregivers’ unfamiliarity with digital technology. These barriers likely reduced participant diversity and introduced selection bias toward individuals more comfortable with technology. Larger, systematically recruited studies are needed to more reliably evaluate clinical effectiveness. Second, as a new application, the system may still have technical limitations and may not yet be user-friendly enough for older adults unfamiliar with digital tools. Baseline cognitive and functional status were also not formally assessed, so these unmeasured factors could have influenced participants’ ability to use the application. Future versions should incorporate structured feedback and usability testing. Third, in-app medication logging is subject to reporting error and behavioral bias. The discordance analysis relied on hospital-derived MPR, which measures pharmacy dispensing rather than actual medication use. Thus, the findings reflect only medication logging behavior. Future studies with laboratory monitoring and BMD assessment are needed for clinical correlation. Fourth, quality-of-life assessment was limited by the absence of standardized instruments such as the EQ-5D or Barthel Index. Lengthy questionnaires were avoided to preserve user engagement, and formal attention checks were deprioritized to reduce cognitive burden on older adults. This was a deliberate design trade-off in the pilot application. As a result, it was not possible to distinguish genuinely high-quality-of-life responses from satisficing. Monthly completion rates for quality of life declined from 100% at baseline to 7.7% at month 12. Additionally, 5 of 26 active responders consistently reported perfect scores at every time point, possibly reflecting high perceived quality of life or nondifferentiated responses. A response rate for the weekly satisfaction prompt could not be calculated because only aggregated monthly satisfaction scores were stored, not individual completion records. Fifth, application interactions were analyzed only as a binary outcome (logging vs nonlogging) rather than by specific interaction types, so detailed engagement trends could not be evaluated. Finally, the 1-year follow-up period is short compared to standard osteoporosis treatment, which usually lasts 3 years or more. Longer-term studies will be needed to assess sustained feasibility, user engagement, application fatigue, and clinical impact across a full treatment course.

Further patient-centered qualitative research is warranted as a next step. Interviews or focus groups could help triangulate the quantitative findings reported in this study, provide deeper insight into the facilitators and barriers affecting sustained application engagement, explore problems or discomfort experienced during real-world use, and better clarify the relationship between application use and medication adherence behaviors.

### Conclusions

The Wellhealth system, including the Assisted Liaison Service feature, was feasible to implement among older adults receiving osteoporosis treatment over a 1-year period. Engagement with daily medication logging prompts was acceptable, with an overall mean logging rate of approximately 60%. In contrast, engagement with the in-app self-assessment surveys was lower, with an overall quality-of-life response rate of 48.1% (200/416), and declined progressively throughout follow-up. Limited technological literacy and inconsistent internet access represented important practical barriers within this cohort. Further refinement of the application, together with larger and longer-term implementation studies, will be necessary to evaluate the system’s clinical effectiveness and long-term impact on treatment management.

## Appendix

Multimedia Appendix 1 Wellhealth Quality of Life Assessment Questions.

## Abbreviations

BMD: bone mineral density
MPR: medication possession ratio

## Data Availability

The datasets generated and/or analyzed during this study are available from the corresponding author upon reasonable request.
