# Supplementary material for: Implementation of a Web-Based Application (Wellhealth) for Osteoporosis Medication Management in Older Adults: Prospective Feasibility Study
Source: JMIR Form Res. 2026 Jun 10;10:e86067. doi: 10.2196/86067 (PMC13294653; doi:10.2196/86067)
Supplement: Multimedia Appendix 1 [file formative_v10i1e86067_app1.docx]

**Well Health Quality of Life Assessment Questions**

There are 5 answer options:

- **1. Not at all (1 point):** You do not feel this way, feel very dissatisfied, or feel very bad.
- **2. A little (2 points):** You rarely feel this way, feel slightly dissatisfied, or feel bad.
- **3. Moderately (3 points):** You feel this way sometimes, feel moderately satisfied, or experience an average level of satisfaction.
- **4. Very much (4 points):** You often feel this way, feel satisfied, or feel good.
- **5. Extremely (5 points):** You always feel this way, feel complete, very satisfied, or very good.

**Questions**

**Section 1: Physical Health**

1. I am satisfied with my physical health.
2. I have enough strength to carry out my daily activities (either in work or in daily life).
3. I am satisfied with my sleep.

**Section 2: Work**

1. I have no concerns about my job and income.
2. I am satisfied with my ability to do my job as I used to.
3. I feel satisfied that I can get things done each day.

**Section 3: Financial**

1. I have no financial concerns from my illness or treatment.
2. I am satisfied with my financial situation and have no concerns about it.
3. I have enough money to spend on necessities.

**Section 4: Mental Health**

1. I feel good, hopeful, positive, happy, and at ease.
2. I accept my current body image and physical appearance.
3. I feel that my life is valuable.

**Section 5: Social**

1. I am satisfied with my friendships or socialization.
2. I am satisfied with the help that I have received from my friends.
3. I am satisfied with my sex life. (Sex life refers to having ways to manage and express sexual feelings, including masturbation or sexual activity.)

**Result Interpretation**

The total score is converted into a percentage. The interpretation criteria are as follows:

- **80–100%: Excellent** quality of life
- **60–79%: Good** quality of life
- **40–59%: Moderate** quality of life
- **20–39%: Fai**r quality of life
- **Below 20%:** Poor quality of life
